# Reinforcement and inference in cross-situational word learning

**DOI:** 10.3389/fnbeh.2013.00163

**Published:** 2013-11-19

**Authors:** Paulo F. C. Tilles, José F. Fontanari

**Affiliations:** Departamento de Física e Informática, Instituto de Física de São Carlos, Universidade de São PauloSão Carlos, Brazil

**Keywords:** statistical learning, word learning, cross-situational learning, associative learning, mutual exclusivity

## Abstract

Cross-situational word learning is based on the notion that a learner can determine the referent of a word by finding something in common across many observed uses of that word. Here we propose an adaptive learning algorithm that contains a parameter that controls the strength of the reinforcement applied to associations between concurrent words and referents, and a parameter that regulates inference, which includes built-in biases, such as mutual exclusivity, and information of past learning events. By adjusting these parameters so that the model predictions agree with data from representative experiments on cross-situational word learning, we were able to explain the learning strategies adopted by the participants of those experiments in terms of a trade-off between reinforcement and inference. These strategies can vary wildly depending on the conditions of the experiments. For instance, for fast mapping experiments (i.e., the correct referent could, in principle, be inferred in a single observation) inference is prevalent, whereas for segregated contextual diversity experiments (i.e., the referents are separated in groups and are exhibited with members of their groups only) reinforcement is predominant. Other experiments are explained with more balanced doses of reinforcement and inference.

## 1. Introduction

A desirable goal of a psychological theory is to offer explanations grounded on elementary principles to the data available from psychology experiments (Newell, 1994). Although most of these quantitative psychological data are related to mental chronometry and memory accuracy, recent explorations on the human performance to acquire an artificial lexicon in controlled laboratory conditions have paved the way to the understanding of the learning strategies humans use to infer a word-object mapping (Yu and Smith, 2007; Kachergis et al., 2009; Smith et al., 2011; Kachergis et al., 2012; Yu and Smith, 2012a). These experiments are based on the cross-situational word-learning paradigm which avers that a learner can determine the meaning of a word by finding something in common across all observed uses of that word (Gleitman, 1990; Pinker, 1990). In that sense, learning takes place through the statistical sampling of the contexts in which a word appears in accord with the classical associationist stance of Hume and Locke that the mechanism of word learning is sensitivity to covariation: if two events occur at the same time, they become associated (Bloom, 2000).

In a typical cross-situational word-learning experiment, participants are exposed repeatedly to multiple unfamiliar objects concomitantly with multiple spoken pseudo-words, such that a word and its correct referent (object) always appear together on a learning trial. Different trials exhibiting distinct word-object pairs will eventually allow the disambiguation of the word-object associations and the learning of the correct mapping (Yu and Smith, 2007). However, it is questionable whether this scenario is suitable to describe the actual word learning process by children even in the unambiguous situation where the single novel object is followed by the utterance of its corresponding pseudo-word. In fact, it was shown that young children will only make the connection between the object and the word provided they have a reason to believe that they are in presence of an act of naming and for this the speaker has to be present (Baldwin et al., 1996; Bloom, 2000; Waxman and Gelman, 2009). Adults could learn those associations either because they were previously instructed by the experimenter that they would be learning which words go with which objects or because they could infer that the disembodied voice is an act of naming by a concealed person. Although there have been claims that cross-situational statistical learning is part of the repertoire of young word learners (Yu and Smith, 2008), the effect of individual differences in attention and vocabulary development of the infants complicates considerably this issue which is still a matter for debate (Yu and Smith, 2012b; Smith and Yu, 2013).

There are several other alternative or complementary approaches to the statistical learning formulation of language acquisition considered in this paper. For instance, the social-pragmatic hypothesis claims that the child makes the connections between words and their referents by understanding the referential intentions of others. This approach, which seems to be originally due to Augustine, implies that children use intuitive psychology to “read” the adults' minds (Bloom, 2000). A more recent approach that explores the grounding of language in perception and action has been proved effective in the design of linguistic capabilities in humanoid cognitive robots (Cangelosi et al., 2007; Cangelosi, 2010; Pezzulo et al., 2013) as well as in the support of word learning by toddlers through the stabilization of their attention on the selected object (Yu and Smith, 2012b). In contrast with the unsupervised cross-situational learning scheme, the scenario known as operant conditioning involves the active participation of the agents in the learning process, with exchange of non-linguistic cues to provide feedback on the learner inferences. This supervised learning scheme has been applied to the design of a system for communication by autonomous robots in the Talking Heads experiments (Steels, 2003). We note that a comparison between the cross-situational and operant conditioning learning schemes indicates that they perform similarly in the limit of very large lexicon sizes (Fontanari and Cangelosi, 2011).

As our goal is to interpret the learning performance of adults using a few plausible reasoning tenets, here we assume that in order to learn a word-object mapping within the cross-situational word-learning scenario the learner should be able to (i) recall at least a fraction of the word-object pairings that appeared in the learning trials, (ii) register both co-occurrences and non-co-occurrences of words and objects and (iii) apply the mutual exclusivity principle which favors the association of novel words to novel objects (Markman and Wachtel, 1988). Of course, we note that a hypothetical learner could achieve cross-situational learning solely by registering and recalling co-occurrences of words and objects without carrying out any inferential reasoning (Blythe et al., 2010; Tilles and Fontanari, 2012a), but we find it implausible that human learners would not reap the benefits (e.g., fast mapping) of employing mutual exclusivity (Vogt, 2012; Reisenauer et al., 2013).

In this paper we offer an adaptive learning algorithm that comprises two parameters which regulate the associative reinforcement of pairings between concurrent words and objects, and the non-associative inference process that handles built-in biases (e.g., mutual exclusivity) as well as information of past learning events. By setting the values of these parameters so as to fit a representative selection of experimental data presented in Kachergis et al. (2009, 2012) we are able to identify and explain the learning strategies adopted by the participants of those experiments in terms of a trade-off between reinforcement and inference.

## 2. Cross-situational learning scenario

We assume there are *N* objects *o*_1_, …, *o_N_*, *N* words *w*_1_, …, *w_N_* and a one-to-one mapping between words and objects represented by the set Γ = {(*w*_1_, *o*_1_), …, (*w_N_*, *o_N_*)}. At each learning trial, *C* word-object pairs are selected from Γ and presented to the learner without providing any clue on which word goes with which object. For instance, pictures of the *C* objects are displayed in a slide while *C* pseudo-words are spoken sequentially such that their spatial and temporal arrangements do not give away the correct word-object associations (Yu and Smith, 2007; Kachergis et al., 2009). We refer to the subset of words and their referents (objects) presented to the learner in a learning trial as the context Ω = {*w*_1_, *o*_1_, *w*_2_, *o*_2_,…, *w_C_*, *o_C_*}. The context size *C* is then a measure of the within-trial ambiguity, i.e., the number of co-occurring word-object pairs per learning trial. The selection procedure from the set Γ, which may favor some particular subsets of word-object pairs, determines the different experimental setups discussed in this paper. Although each individual trial is highly ambiguous, repetition of trials with partially overlapping contexts should in principle allow the learning of the *N* word-object associations.

After the training stage is completed, which typically comprises about two dozen trials, the learning accuracy is measured by instructing the learner to pick the object among the *N* objects on display which the learner thinks is associated to a particular target word. The test is repeated for all *N* words and the average learning accuracy calculated as the fraction of correct guesses (Kachergis et al., 2009).

This cross-situational learning scenario does not account for the presence of noise, such as the effect of out-of-context words. This situation can be modeled by assuming that there is a certain probability (noise) that the referent of one of the spoken words is not part of the context (so that word can be said to be out of context). Although theoretical analysis shows that there is a maximum noise intensity beyond which statistical learning is unattainable (Tilles and Fontanari, 2012b), as yet no experiment was carried out to verify the existence of this threshold phenomenon on the learning performance of human subjects.

## 3. Model

We model learning as a change in the confidence with which the algorithm (or, for simplicity, the learner) associates the word *w*_*i*_ to an object *o*_*j*_ that results from the observation and analysis of the contexts presented in the learning trials. More to the point, this confidence is represented by the probability *P*_*t*_(*w*_*i*_, *o*_*j*_) that *w*_*i*_ is associated to *o*_*j*_ at learning trial *t*. This probability is normalized such that ∑_o_*j*__ *P*_*t*_(*w*_*i*_, *o*_*j*_) = 1 for all *w*_*i*_ and *t* > 0, which then implies that when the word *w*_*i*_ is presented to the learner in the testing stage the learning accuracy is given simply by *P*_*t*_(*w*_*i*_, *o*_*i*_). In addition, we assume that *P*_*t*_(*w*_*i*_, *o*_*j*_) contains information presented in the learning trials up to and including trial *t* only.

If at learning trial *t* the learner observes the context Ω_*t*_ = {*w*_1_, *o*_1_, *w*_2_, *o*_2_,…, *w_C_*, *o_C_*} then it can infer the existence of two other informative sets. First, the set of the words (and their referents) that appear for the first time at trial *t*, which we denote by ${\tilde{\Omega }}_{t}=\left\{{\tilde{w}}_{1},{\tilde{o}}_{1},{\tilde{w}}_{2},{\tilde{o}}_{2},\ldots ,{\tilde{w}}_{\tilde{C}},{\tilde{o}}_{{\tilde{C}}_{t}}\right\}$. Clearly, ${\tilde{\Omega }}_{t}\subseteq \Omega_{t}$ and ${\tilde{C}}_{t}\leq C$. Second, the set of words (and their referents) that do not appear in Ω_*t*_ but that have already appeared in the previous trials, ${\bar{\Omega }}_{t}=\left\{{\bar{w}}_{1},{\bar{o}}_{1},\ldots ,{\bar{w}}_{N_{t}-C},{\bar{o}}_{N_{t}-C}\right\}$ where *N*_*t*_ is the total number of different words that appeared in contexts up to and including trial *t*. Clearly, ${\bar{\Omega }}_{t}\cap \Omega_{t}=\emptyset$. The update rule of the confidences *P*_*t*_(*w*_*i*_, *o*_*j*_) depends on which of these three sets the word *w*_*i*_ and the object *o*_*j*_ belong to (if *i* ≠ *j* they may belong to different sets). In fact, our learning algorithm comprises a parameter χ ∈ [0,1] that measures the associative reinforcement capacity and applies only to known words that appear in the current context, and a parameter β ∈ [0,1] that measures the inference capacity and applies either to known words that do not appear in the current context or to new words in the current context. Before the experiment begins (*t* = 0) we set *P*_0_ (*w*_*i*_, *o*_*j*_) = 0 for all words *w*_*i*_ and objects *o*_*j*_. Next we describe how the confidences are updated following the sequential presentation of contexts.

In the first trial (*t* = 1) all words are new (${\tilde{C}}_{1}$ = *N*_1_ = *C*), so we set

(1) $$P_{1}\left({\tilde{w}}_{i},{\tilde{o}}_{j}\right)=\frac{1}{C}$$

for ${\tilde{w}}_{i}$,${\tilde{o}}_{j}$ ∈ $\tilde{\Omega }$ = Ω. In the second or in an arbitrary trial *t* we expect to observe contexts exhibiting both novel and repeated words. Novel words must go through an inference preprocessing stage before the reinforcement procedure can be applied to them. This is so because if ${\tilde{w}}_{i}$ appears for the first time at trial *t* then *P*_*t* − 1_ (${\tilde{w}}_{i}$, *o*_*j*_) = 0 for all objects *o*_*j*_ and since the reinforcement is proportional to *P*_*t* − 1_ (${\tilde{w}}_{i}$, *o*_*j*_) the confidences associated to ${\tilde{w}}_{i}$ would never be updated (see Equation 5 and the explanation thereafter). Thus, when a novel word ${\tilde{w}}_{i}$ appear at trial *t* ≥ 2, we redefine its confidence values at the previous trial (originally set to zero) as

(2) $$P_{t-1}\left({\tilde{w}}_{i},{\tilde{o}}_{j}\right)=\frac{\beta }{{\tilde{C}}_{t}}+\frac{1-\beta }{N_{t-1}+{\tilde{C}}_{t}},$$

(3) $$P_{t-1}\left({\tilde{w}}_{i},o_{j}\right)=\frac{1-\beta }{N_{t-1}+{\tilde{C}}_{t}},$$

(4) $$P_{t-1}\left({\tilde{w}}_{i},{\bar{o}}_{j}\right)=\frac{1-\beta }{N_{t-1}+{\tilde{C}}_{t}}.$$

On the one hand, setting the inference parameter β to its maximum value β = 1 enforces the mutual exclusivity principle which requires that the new word ${\tilde{w}}_{i}$ be associated with equal probability to the ${\tilde{C}}_{t}$ new objects ${\tilde{o}}_{j}$ in the current context. Hence in the case ${\tilde{C}}_{t}$ = 1 the meaning of the new word would be inferred in a single presentation. On the other hand, for β = 0 the new word is associated with equal probability to all objects already seen up to and including trial *t*, i.e., *N*_*t*_ = *N*_*t* − 1_ + ${\tilde{C}}_{t}$. Intermediate values of β describe a situation of imperfect inference. Note that using Equations 2–4 we can easily verify that $\sum_{{\tilde{o}}_{j}}P_{t\;-\;1}\left({\tilde{w}}_{i},{\tilde{o}}_{j}\right)+\sum_{o_{j}}P_{t\;-\;1}\left({\tilde{w}}_{i},o_{j}\right)+\sum_{{\bar{o}}_{j}}P_{t\;-\;1}\left({\tilde{w}}_{i},{\bar{o}}_{j}\right)=1$, in accord with the normalization constraint.

Now we can focus on the update rule of the confidence *P*_*t*_ (*w*_*i*_, *o*_*j*_) in the case both word *w*_*i*_ and object *o*_*j*_ appear in the context at trial *t*. The rule applies both to repeated and novel words, provided the confidences of the novel words are preprocessed according to Equations 2–4. In order to fulfill automatically the normalization condition for word *w*_*i*_, the increase of the confidence *P*_*t*_ (*w*_*i*_, *o*_*j*_) with *o*_*j*_ ∈ Ω_*t*_ must be compensated by the decrease of the confidences *P*_*t*_ (*w*_*i*_, $\bar{o}$_*j*_) with $\bar{o}$_*j*_ ∈ $\bar{\Omega }$_*t*_. This can be implemented by distributing evenly the total flux of probability out of the latter confidences, i.e., $\sum_{{\bar{o}}_{j}\in {\bar{\Omega }}_{t}}P_{t\;-\;1}\left(w_{i},{\bar{o}}_{j}\right)$, over the confidences *P*_*t*_ (*w*_*i*_, *o*_*j*_) with *o*_*j*_ ∈ Ω_*t*_. Hence the net gain of confidence on the association between *w*_*i*_ and *o*_*j*_ is given by

(5) $$r_{t-1}\text{​}\left(w_{i},o_{j}\right)=\chi P_{t-1}\text{​}\left(w_{i},o_{j}\right)\frac{\sum_{{\bar{o}}_{j}\in {\bar{\Omega }}_{t}}P_{t-1}\text{​}\left(w_{i},{\bar{o}}_{j}\right)}{\sum_{o_{j}\in \Omega_{t}}P_{t-1}\text{​}\left(w_{i},o_{j}\right)}$$

where, as mentioned before, the parameter χ ∈ [ 0, 1] measures the strength of the reinforcement process. Note that if both *o*_*j*_ and *o*_*k*_ appear in the context together with *w*_*i*_ then the reinforcement procedure should not create any distinction between the associations (*w*_*i*_, *o*_*j*_) and (*w*_*i*_, *o*_*k*_). This result is achieved provided that the ratio of the confidence gains equals the ratio of the confidences before reinforcement, i.e., *r*_*t* − 1_(*w*_*i*_, *o*_*j*_)/*r*_*t* − 1_(*w*_*i*_, *o*_*k*_) = *P*_*t* − 1_(*w*_*i*_, *o*_*j*_)/*P*_*t* − 1_(*w*_*i*_, *o*_*k*_). This is the reason that the reinforcement gain of a word-object association given by Equation 5 is proportional to the previous confidence on that association. The total increase in the confidences between *w*_*i*_ and the objects that appear in the context, i.e., ∑_*o*_*j*_ ∈ Ω_*t*__ *r*_*t* − 1_(*w*_*i*_, *o*_*j*_), equals the product of χ and the total decrease in the confidences between *w*_*i*_ and the objects that do not appear in the context, i.e., $\sum_{{\bar{o}}_{j}\in {\bar{\Omega }}_{t}}P_{t\;-\;1}\left(w_{i},{\bar{o}}_{j}\right)$. So for χ = 1 the confidences associated to objects absent from the context are fully transferred to the confidences associated to objects present in the context. Lower values of χ allows us to control the flow of confidence from objects in $\bar{\Omega }$_*t*_ to objects in Ω_*t*_.

Most importantly, in order to implement the reinforcement process the learner should be able to gauge the relevance of the information about the previous trials, which is condensed on the confidence values *P*_*t*_(*w*_*i*_, *o*_*j*_). The gauging of this information is quantified by the word and trial dependent quantity α_*t*_(*w*_*i*_) ∈ [ 0, 1] that allows for the interpolation between the cases of maximum relevance (α_*t*_ (*w*_*i*_) = 1) and complete irrelevancy (α_*t*_ (*w*_*i*_) = 0) of the information stored in the confidences *P*_*t*_ (*w*_*i*_, *o*_*j*_). In particular, we assume that the greater the certainty on the association between word *w*_*i*_ and its referent, the more relevant that information is to the learner. A quantitative measure of the uncertainty associated to the confidences regarding word *w*_*i*_ is given by the entropy

(6) $$H_{t}\text{​}\left(w_{i}\right)=-\sum_{o_{j}\in \Omega_{t}\cup {\bar{\Omega }}_{t}}P_{t}\text{​}\left(w_{i},o_{j}\right)\log\left[P_{t}\text{​}\left(w_{i},o_{j}\right)\right]$$

whose maximum (log *N*_*t*_) is obtained by the uniform distribution *P*_*t*_(*w*_*i*_, *o*_*j*_) = 1/*N*_*t*_ for all *o*_*j*_ ∈ Ω_*t*_ ∪ $\bar{\Omega }$_*t*_, and whose minimum (0) by *P*_*t*_(*w*_*i*_, *o*_*j*_) = 1 and *P*_*t*_(*w*_*i*_, *o*_*k*_) = 0 for *o*_*k*_ ≠ *o*_*j*_. So we define

(7) $$\alpha_{t}\text{​}\left(w_{i}\right)=\alpha_{0}+\left(1-\alpha_{0}\right)\left[1-\frac{H_{t}\text{​}\left(w_{i}\right)}{\log N_{t}}\right],$$

where α_0_ ∈ [ 0, 1] is a baseline information gauge factor corresponding to the maximum uncertainty about the referent of a target word.

Finally, recalling that at trial *t* the learner has access to the sets Ω_*t*_, $\bar{\Omega }$_*t*_ as well as to the confidences at trial *t* − 1 we write the update rule

(8) $$P_{t}\text{​}\left(w_{i},o_{j}\right)=P_{t-1}\text{​}\left(w_{i},o_{j}\right)+\alpha_{t-1}\text{​}\left(w_{i}\right)\text{​}r_{t-1}\left(w_{i},o_{j}\right)+\left[1-\alpha_{t-1}\left(w_{i}\right)\right]\left[\frac{1}{N_{t}}-P_{t-1}\left(w_{i},o_{j}\right)\right]$$

for *w*_*i*_, *o*_*j*_ ∈ Ω_*t*_. Note that if α_*t* − 1_(*w*_*i*_) = 0 the learner would associate word *w*_*i*_ to all objects that have appeared up to and including trial *t* with equal probability. This situation happens only if α_0_ = 0 and if there is complete uncertainty about the referent of word *w*_*i*_. Hence the quantity α_*t*_(*w*_*i*_) determines the extent to which the previous confidences on associations involving word *w*_*i*_ influence the update of those confidences.

Now we consider the update rule for the confidence *P*_*t*_ (*w*_*i*_, $\bar{o}$_*j*_) in the case that word *w*_*i*_ appears in the context at trial *t* but object $\bar{o}$_*j*_ does not. (We recall that object $\bar{o}$_*j*_ must have appeared in some previous trial.) According to the reasoning that led to Equation 5 this confidence must decrease by the amount χ *P*_*t* − 1_ (*w*_*i*_, $\bar{o}$_*j*_) and so, taking into account the information gauge factor, we obtain

(9) $$P_{t}\text{​}\left(w_{i},{\bar{o}}_{j}\right)=P_{t-1}\text{​}\left(w_{i},{\bar{o}}_{j}\right)-\alpha_{t-1}\text{​}\left(w_{i}\right)\chi P_{t-1}\text{​}\left(w_{i},{\bar{o}}_{j}\right)+\left[1-\alpha_{t-1}\text{​}\left(w_{i}\right)\right]\text{​}\left[\frac{1}{N_{t}}-P_{t-1}\text{​}\left(w_{i},{\bar{o}}_{j}\right)\right]$$

which can be easily seen to satisfy the normalization

(10) $$\sum_{o_{j}\in \Omega_{t}}P_{t}\text{​}\left(w_{i},o_{j}\right)+\sum_{{\bar{o}}_{j}\in {\bar{\Omega }}_{t}}P_{t}\text{​}\left(w_{i},{\bar{o}}_{j}\right)=1.$$

We focus now on the update rule for the confidence *P*_*t*_ ($\bar{w}$_*i*_, $\bar{o}$_*j*_) with $\bar{w}$_*i*_, $\bar{o}$_*j*_ ∈ $\bar{\Omega }$_*t*_, i.e., both the word $\bar{w}$_*i*_ and the object $\bar{o}$_*j*_ are absent from the context shown at trial *t*, but they have already appeared, not necessarily together, in previous trials. A similar inference reasoning that led to the expressions for the preprocessing of new words would allow the learner to conclude that a word absent from the context should be associated to an object that is also absent from it. In that sense, confidence should flow from the associations between $\bar{w}$_*i*_ and objects *o*_*j*_ ∈ Ω_*t*_ to the associations between $\bar{w}$_*i*_ and objects $\bar{o}$_*j*_ ∈ $\bar{\Omega }$_*t*_. Hence, ignoring the information gauge factor for the moment, the net gain to confidence *P*_*t*_($\bar{w}$_*i*_, $\bar{o}$_*j*_) is given by

(11) $${\bar{r}}_{t-1}\text{​}\left({\bar{w}}_{i},{\bar{o}}_{j}\right)=\beta P_{t-1}\text{​}\left({\bar{w}}_{i},{\bar{o}}_{j}\right)\frac{\sum_{o_{j}\in \Omega_{t}}P_{t-1}\text{​}\left({\bar{w}}_{i},o_{j}\right)}{\sum_{{\bar{o}}_{j}\in {\bar{\Omega }}_{t}}P_{t-1}\text{​}\left({\bar{w}}_{i},{\bar{o}}_{j}\right)}.$$

The direct proportionality of this gain to *P*_*t* − 1_($\bar{w}$_*i*_, $\bar{o}$_*j*_) can be justified by an argument similar to that used to justify Equation 5 in the case of reinforcement. The information relevance issue is also handled in a similar manner so the desired update rule reads

(12) $$P_{t}\text{​}\left({\bar{w}}_{i},{\bar{o}}_{j}\right)=P_{t-1}\text{​}\left({\bar{w}}_{i},{\bar{o}}_{j}\right)+\alpha_{t-1}\text{​}\left({\bar{w}}_{i}\right){\bar{r}}_{t-1}\text{​}\left({\bar{w}}_{i},{\bar{o}}_{j}\right)\text{​​}+\left[1-\alpha_{t-1}\text{​}\left({\bar{w}}_{i}\right)\right]\text{​}\left[\frac{1}{N_{t}}-P_{t-1}\text{​}\left({\bar{w}}_{i},{\bar{o}}_{j}\right)\right]$$

for $\bar{w}$_*i*_, $\bar{o}$_*j*_ ∈ $\bar{\Omega }$_*t*_. To ensure normalization the confidence *P*_*t*_ ($\bar{w}$_*i*_, *o*_*j*_) must decrease by an amount proportional to β*P*_*t* − 1_($\bar{w}$_*i*_, *o*_*j*_) so that

(13) $$P_{t}\text{​}\left({\bar{w}}_{i},o_{j}\right)=P_{t-1}\text{​}\left({\bar{w}}_{i},o_{j}\right)-\alpha_{t-1}\text{​}\left({\bar{w}}_{i}\right)\beta P_{t-1}\text{​}\left({\bar{w}}_{i},o_{j}\right)\text{​​}+\left[1-\alpha_{t-1}\text{​}\left({\bar{w}}_{i}\right)\right]\text{​}\left[\frac{1}{N_{t}}-P_{t-1}\text{​}\left({\bar{w}}_{i},o_{j}\right)\right]$$

for $\bar{w}$_*i*_ ∈ $\bar{\Omega }$_*t*_ and *o*_*j*_ ∈ Ω_*t*_. We can verify that prescriptions (12) and (13) satisfy the normalization

(14) $$\sum_{{\bar{o}}_{j}\in {\bar{\Omega }}_{t}}P_{t}\text{​}\left({\bar{w}}_{i},{\bar{o}}_{j}\right)+\sum_{o_{j}\in \Omega_{t}}P_{t}\text{​}\left({\bar{w}}_{i},o_{j}\right)=1,$$

as expected.

In summary, before any trial (*t* = 0) we set all confidence values to zero, i.e., *P*_0_(*w*_*i*_, *o*_*j*_) = 0, and fix the values of the parameters α_0_, χ and β. In the first trial (*t* = 1) we set the confidences of the words and objects in Ω_1_ according to Equation (1), so we have the values of *P*_1_(*w*_*i*_, *o*_*j*_) for w_*i*_, *o*_*j*_ ∈ Ω_1_. In the second trial, we separate the novel words ${\tilde{w}}_{i}$ ∈ ${\tilde{\Omega }}_{2}$ and reset *P*_1_(${\tilde{w}}_{i}$, *o*_*j*_) with *o*_*i*_ ∈ Ω_2_ ∪ $\bar{\Omega }$_2_ according to Equations 2–4. Only then we calculate α_1_(*w*_*i*_) with *w*_*i*_ ∈ Ω_1_ ∪ ${\tilde{\Omega }}_{2}$ using Equation (7). The confidences at trial *t* = 2 then follows from Equations (8), (9), (12), and (13). As before, in the third trial we separate the novel words ${\tilde{w}}_{i}$ ∈ ${\tilde{\Omega }}_{3}$, reset *P*_2_(${\tilde{w}}_{i}$, *o*_*j*_) with *o*_*i*_ ∈ Ω_3_ ∪ $\bar{\Omega }$_3_ according to Equations 2–4, calculate α_2_(*w*_*i*_) with *w*_*i*_ ∈ Ω_1_ ∪ Ω_2_ ∪ ${\tilde{\Omega }}_{3}$ using Equation (7), and only then resume the evaluation of the confidences at trial *t* = 3. This procedure is repeated until the training stage is completed, say, at *t* = t^*^. At this point, knowledge of the confidence values *P*_t^*^_ (*w*_*i*_, *o*_*j*_) allows us to answer any question posed in the testing stage.

Our model borrows many features from other proposed models of word learning (Siskind, 1996; Fontanari et al., 2009; Frank et al., 2009; Fazly et al., 2010; Kachergis et al., 2012). In particular, the entropy expression (6) was used by Kachergis et al. (2012) to allocate attention trial-by-trail to the associations presented in the contexts. Here we use that expression to quantify the uncertainty associated to the various confidences in order to determine the extent to which those confidences are updated on a learning trial. A distinctive feature of our model is the update of associations that are not in the current trial according to Equation (12). In particular, we note that whereas *ad hoc* normalization can only decrease the confidences on associations between words and objects that did not appear in the current context, our update rule can increase those associations as well. The extent of this update is weighted by the inference parameter β and it allows the application of mutual exclusivity to associations that are not shown in the current context. In fact, the splitting of mental processes in two classes, namely, reinforcement processes that update associations in the current context and inference processes that update the other associations is the main thrust of our paper. In the next section we evaluate the adequacy of our model to describe a selection of cross-situational word-learning experiments carried out on adult subjects by Kachergis et al. (2009, 2012).

## 4. Results

The cross-situational word-learning experiments of Kachergis et al. (2009, 2012) aimed to understand how word sampling frequency (i.e., number of trials in which a word appears), contextual diversity (i.e., the co-occurrence of distinct words or groups of words in the learning trials), within-trial ambiguity (i.e., the context size *C*), and fast-mapping of novel words affect the learning performance of adult subjects. In this section we compare the performance of the algorithm described in the previous section with the performance of adult subjects reported in Kachergis et al. (2009, 2012). In particular, once the conditions of the training stage are specified, we carry out 10^4^ runs of our algorithm for fixed values of the three parameters α_0_, β, χ, and then calculate the average accuracy at trial *t* = t^*^ over all those runs for that parameter setting. Since the algorithm is deterministic, what changes in each run is the composition of the contexts at each learning trial. As our goal is to model the results of the experiments, we search the space of parameters to find the setting such that the performance of the algorithm matches that of humans within the error bars (i.e., one standard deviation) of the experiments.

### 4.1. Word sampling frequency

In these experiments the number of words (and objects) is *N* = 18 and the training stage totals t^*^ = 27 learning trials, with each trial comprising the presentation of 4 words together with their referents (*C* = 4). Following Kachergis et al. (2009), we investigate two conditions which differ with respect to the number of times a word is exhibited in the training stage. In the two-frequency condition, the 18 words are divided into two subsets of 9 words each. The words in the first subset appear 9 times and those in the second only 3 times. In the three-frequency condition, the 18 words are divided into three subsets of 6 words each. Words in the first subset appear 3 times, in the second, 6 times and in the third, 9 times. In these two conditions, the same word was not allowed to appear in two consecutive learning trials.

**Figure 1:**
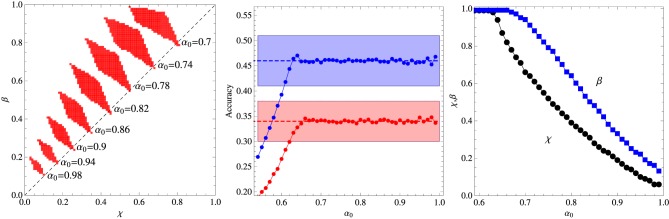
**Summary of the results for the two-frequency condition experiment. Left panel:** Regions in the plane (χ, β) where the algorithm fits the experimental data for fixed α_0_ as indicated in the figure. **Middle panel:** Average accuracy for the best fit to the results of Experiment 1 of Kachergis et al. (2009) represented by the broken horizontal lines (means) and shaded regions around them (one standard deviation). The blue symbols represent the accuracy for the group of words sampled 9 times whereas the red symbols represent the accuracy for the words sampled 3 times. **Right panel:** Parameters χ and β corresponding to the best fit shown in the middle panel. The other parameters are *N* = 18 and *C* = 4.

**Figure 2:**
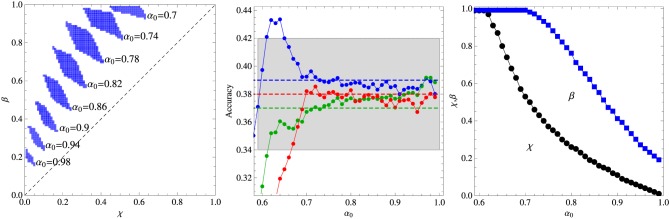
**Summary of the results for the three-frequency condition experiment. Left panel:** Regions in the plane (χ, β) where the algorithm fits the experimental data for fixed α_0_ as indicated in the figure. **Middle panel:** Average accuracy for the best fit to the results of Experiment 1 of Kachergis et al. (2009) represented by the broken horizontal lines (means) and shaded regions around them (one standard deviation). The blue symbols represent the accuracy for the group of words sampled 9 times, the green symbols for the words sampled 6 times, and the red symbols for the words sampled 3 times. **Right panel:** Parameters χ and β corresponding to the best fit shown in the middle panel. The other parameters are *N* = 18 and *C* = 4.

Figures 1, 2 summarize our main results for the two-frequency and three-frequency conditions, respectively. The left panels show the regions (shaded areas) in the (χ, β) plane for fixed α_0_ where the algorithm describes the experimental data. We note that if those regions are located left to the diagonal χ = β then the inference process is dominant whereas if they are right to the diagonal then reinforcement is the dominant process. The middle panels show the accuracy of the best fit as function of the parameter α_0_ and the right panels exhibit the values of χ and β corresponding to that fit. The broken horizontal lines and the shaded zones around them represent the means and standard deviations of the results of experiments carried out with 33 adult subjects (Kachergis et al., 2009).

It is interesting that although the words sampled more frequently are learned best in the two-frequency condition as expected, this advantage practically disappears in the three-frequency condition in which case all words are learned at equal levels within the experimental error. Note that the average accuracy for the words sampled 3 times is actually greater than the accuracy for the words sampled 6 times, but this inversion is not statistically significant, although, most surprisingly, the algorithm does reproduce it for α_0_ ∈ [0.7, 0.8]. According to Kachergis et al. (2009), the reason for the observed sampling frequency insensitivity might be because the high-frequency words are learned quickly and once they are learned subsequent trials containing those words will exhibit an effectively smaller within-trial ambiguity. In this vein, the inversion could be explained if by chance the words less frequently sampled were generally paired with the highly sampled words. Thus, contextual diversity seems to play a key role in cross-situational word learning.

### 4.2. Contextual diversity and within-trial ambiguity

**Figure 3:**
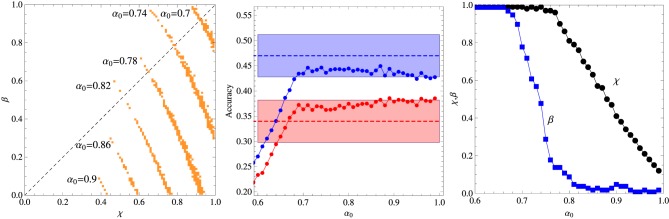
**Summary of the results of the two-level contextual diversity experiment. Left panel:** Regions in the plane (χ, β) where the algorithm fits the experimental data for fixed α_0_ as indicated in the figure. **Middle panel:** Average accuracy for the best fit to the results of Experiment 2 of Kachergis et al. (2009) represented by the broken horizontal lines (means) and shaded regions around them (one standard deviation). The blue symbols represent the accuracy for the group of words belonging to the 12-components subgroup and the red symbols for the words belonging to the 6-components subgroup. All words are repeated exactly 6 times during the t^*^ = 27 learning trials. **Right panel:** Parameters χ and β corresponding to the best fit shown in the middle panel. The other parameters are *N* = 18 and *C* = 3.

In the first experiment aiming to probe the role of contextual diversity in the cross-situational learning, the 18 words were divided in two groups of 6 and 12 words each, and the contexts of size *C* = 3 were formed with words belonging to the same group only. Since the sampling frequency was fixed to 6 repetitions for each word, those words belonging to the more numerous group are exposed to a larger contextual diversity (i.e., the variety of different words with which a given word appear in the course of the training stage). The results summarized in Figure 3 indicate clearly that contextual diversity enhances the learning accuracy. Perhaps more telling is the finding that incorrect responses are largely due to misassignments to referents whose words belong to the same group of the test word. In particular, Kachergis et al. (2009) found that this type of error accounts for 56% of incorrect answers when the test word belongs to the 6-components subgroup and for 76% when it belongs to the 12-components subgroup. The corresponding statistics for our algorithm with the optimal parameters set at α_0_ = 0.9 are 43% and 70%, respectively. The region in the space of parameters where the model can be said to describe the experimental data is greatly reduced in this experiment and even the best fit is barely within the error bars. It is interesting that, contrasting with the previous experiments, in this case the reinforcement procedure seems to play the more important role in the performance of the algorithm.

**Figure 4:**
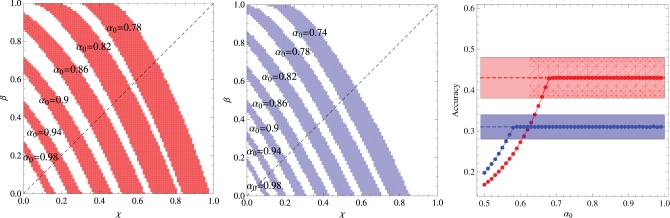
Summary of the results of the experiments where all words co-occur without constraint and the *N* = 18 words are repeated exactly 6 times during the t^*^ = 27 learning trials. Left panel: Regions in the plane (χ, β) where the algorithm fits the experimental data for fixed α_0_ and context size *C* = 3. **Middle panel:** Same as the left panel but for context size *C* = 4. **Right panel:** Average accuracy for the best fitting of the results of Experiment 2 of Kachergis et al. (2009) represented by the broken horizontal lines (means) and shaded regions around them (one standard deviation). The red symbols are for *C* = 3 and the blue symbols for *C* = 4.

The effect of the context size or within-trial ambiguity is addressed by the experiment summarized in Figure 4, which is similar to the previous experiment, except that the words that compose the context are chosen uniformly from the entire repertoire of *N* = 18 words. Two context sizes are considered, namely, *C* = 3 and *C* = 4. In both cases, there is a large selection of parameter values that explain the experimental data, yielding results indistinguishable from the experimental average accuracies. This is the reason we do not exhibit a graph akin to those shown in the right panels of the previous figures. Since a perfect fitting can be obtained both for χ > β and for χ < β, this experiment is uninformative with respect to these two abilities. As expected, increase of the within-trial ambiguity difficilitate learning. In addition, the (experimental) results for *C* = 3 yield a learning accuracy value that is intermediary to those measured for the 6 and 12-components subgroups, which is in agreement with the conclusion that the increase of the contextual diversity enhances learning, since the mean number of different co-occurring words is 4.0 in the 6-components subgroup, 9.2 in the 12-components subgroup and 8.8 in the uniformly mixed situation (Kachergis et al., 2009).

### 4.3. Fast mapping

The experiments carried out by Kachergis et al. (2012) were designed to elicit participants' use of the mutual exclusivity principle (i.e., the assumption of one-to-one mappings between words and referents) and to test the flexibility of a learned word-object association when new evidence is provided in support to a many-to-many mapping. To see how mutual exclusivity implies fast mapping assume that a learner who knows the association (*w*_1_, *o*_1_) is exposed to the context Ω = {*w*_1_, *o*_1_, *w*_2_, *o*_2_} in which the word *w*_2_ (and its referent) appears for the first time. Then it is clear that a mutual-exclusivity-biased learner would infer the association (*w*_2_, *o*_2_) in this single trial. However, a purely associative learner would give equal weights to *o*_1_ and *o*_2_ if asked about the referent of *w*_2_.

In the specific experiment we address in this section, *N* = 12 words and their referents are split up into two groups of 6 words each, say *A* = {(*w*_1_, *o*_1_), …, (*w*_6_, *o*_6_)} and *B* = {(*w*_7_, *o*_7_), …, (*w*_12_, *o*_12_)}. The context size is set to *C* = 2 and the training stage is divided in two phases. In the early phase, only the words belonging to group *A* are presented and the duration of this phase is set such that each word is repeated 3, 6 or 9 times. In the late phase, the contexts consist of one word belonging to *A* and one belonging to *B* forming fixed couples, i.e., whenever *w*_*i*_ appears in a context, *w*_*i* + 6_, with *i* = 1, …, 6, must appear too. The duration of the late phase depends on the number of repetitions of each word that can be 3, 6, or 9 as in the early phase (Kachergis et al., 2012). The combinations of the sampling frequencies yield 9 different training conditions but here we will consider only the case that the late phase comprises 6 repetitions of each word.

**Figure 5:**
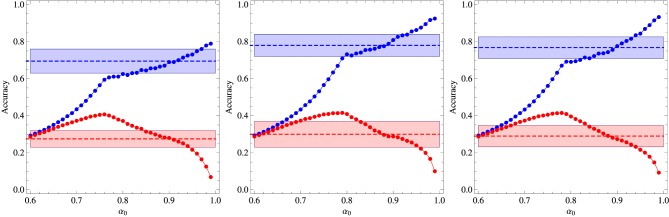
**Results of the experiments on mutual exclusivity in the case the late phase of the training process comprises 6 repetitions of each word**. The blue symbols represent the probability that the algorithm picks object *o*_1_ as the referent of word *w*_1_ whereas the red symbols represent the probability it picks *o*_7_. The broken horizontal lines and the shaded zones around them represent the experimental means and standard deviations (Kachergis et al., 2012) represented by the broken horizontal lines (means) and shaded regions around them (one standard deviation). The left panel shows the results for 3 repetitions of *w*_1_ in the early training phase, the middle panel for 6 repetitions and the right panel for 9 repetitions. The results correspond to the parameters χ and β that best fit the experimental data for fixed α_0_.

**Figure 6:**
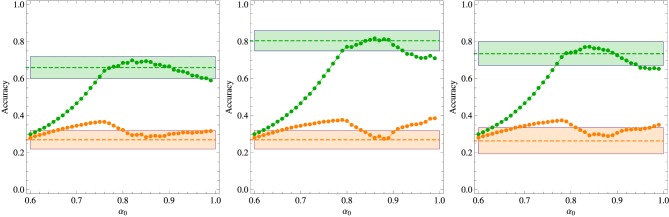
**Results of the experiments on mutual exclusivity in the case the late phase of the training process comprises 6 repetitions of each word**. The green symbols represent the probability that the algorithm picks object *o*_7_ as the referent of word *w*_7_ whereas the orange symbols represent the probability it picks *o*_1_. The broken horizontal lines and the shaded zones around them represent the experimental means and standard deviations (Kachergis et al., 2012) represented by the broken horizontal lines (means) and shaded regions around them (one standard deviation). The left panel shows the results for 3 repetitions of *w*_1_ in the early training phase, the middle panel for 6 repetitions and the right panel for 9 repetitions. The results correspond to the parameters χ and β that best fit the experimental data for fixed α_0_.

**Figure 7:**
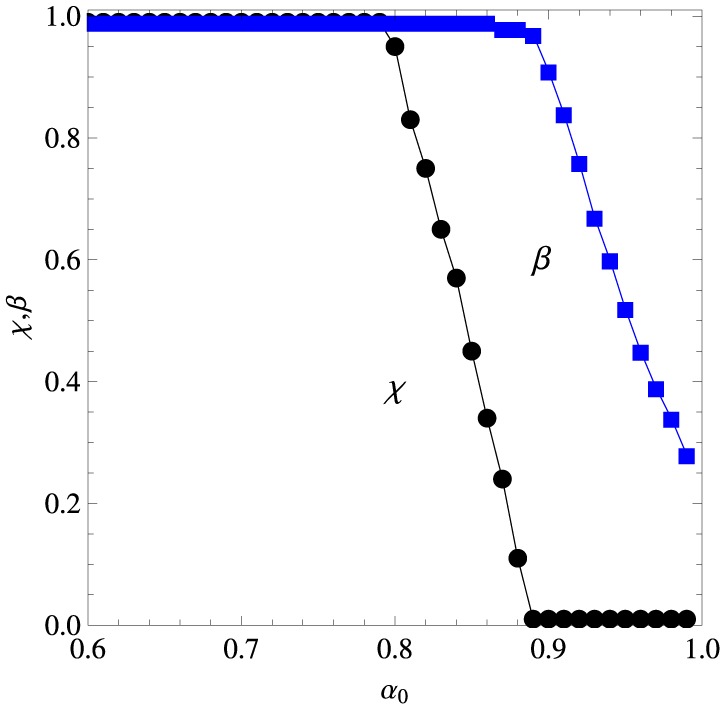
**Parameters χ (reinforcement strength) and β (inference strength) corresponding to the best fit shown in Figures 5 and 6 in the case word *w*_1_ is repeated 6 times in the early training phase**.

The testing stage comprises the play of a single word, say *w*_1_, and the display of 11 of the 12 trained objects (Kachergis et al., 2012). Each word was tested twice with a time lag between the tests: once without its corresponding early object (*o*_1_ in the case) and once without its late object (*o*_7_ in the case). This procedure requires that we renormalize the confidences for each test. For instance, in the case *o*_1_ is left out of the display, the renormalization is

(15) $$P_{t^{*}}\prime \text{​}\left(w_{1},o_{j}\right)=P_{t^{*}}\text{​}\left(w_{1},o_{j}\right)/\sum_{o_{k}\neq o_{1}}P_{t^{*}}\text{​}\left(w_{1},o_{k}\right)$$

with *j* = 2, …, 12 so that ∑_*o*_*j*_ ≠ *o*_1__ *P*_t^*^_′ (*w*_1_, *o*_*j*_) = 1. Similarly, in the case *o*_7_ is left out the renormalization becomes

(16) $$P_{t^{*}}\prime \text{​}\left(w_{1},o_{j}\right)=P_{t^{*}}\text{​}\left(w_{1},o_{j}\right)/\sum_{o_{k}\neq o_{7}}P_{t^{*}}\text{​}\left(w_{1},o_{k}\right)$$

with *j* = 1, …, 6, 8,…, 12 so that ∑_*o*_*j*_ ≠ *o*_7__ *P*_t^*^_′ (*w*_1_, *o*_*j*_) = 1. We are interested on the (renormalized) confidences *P*_t^*^_′ (*w*_1_, *o*_1_), *P*_t^*^_′ (*w*_1_, *o*_7_), *P*_t^*^_′ (*w*_7_, *o*_7_), and *P*_t^*^_′ (*w*_7_, *o*_1_), which are shown in Figures 5, 6 for the conditions where words *w*_*i*_, *i* = 1, …,6 are repeated 3 (left panel), 6 (middle panel), and 9 (right panel) times in the early learning phase, and the words *w*_*i*_, *i* = 1, …,12 are repeated 6 times in the late phase. The figures exhibit the performance of the algorithm for the set of parameters χ and β that fits best the experimental data of Kachergis et al. (2012) for fixed α_0_. This optimum set is shown in Figure 7 for the 6 early repetition condition, which is practically indistinguishable from the optima of the other two conditions. The conditions with the different word repetitions in the early phase intended to produce distinct confidences on the learned association (*w*_1_, *o*_1_) before the onset of the late phase in the training stage. The insensitivity of the results to these conditions probably indicates that association was already learned well enough with 3 repetitions only. Finally, we note that, though the testing stage focused on words *w*_1_ and *w*_7_ only, all word pairs *w*_*i*_ and *w*_*i*+6_ with *i* = 1,…,6 are strictly equivalent since they appear the same number of times during the training stage.

The experimental results exhibited in Figure 6 offer indirect evidence that the participants have resorted to mutual exclusivity to produce their word-object mappings. In fact, from the perspective of a purely associative learner, word *w*_7_ should be associated to objects *o*_1_ or *o*_7_ only, but since in the testing stage one of those objects was not displayed, such a learner would surely select the correct referent. However, the finding that *P*_t^*^_′ (*w*_7_, *o*_7_) is considerably greater than *P*_t^*^_′ (*w*_7_, *o*_1_) (they should be equal for an associative learner) indicates that there is a bias against the association (*w*_7_, *o*_1_) which is motivated, perhaps, from the previous understanding that *o*_1_ was the referent of word *w*_1_. In fact, a most remarkable result revealed by Figure 6 is that *P*_t^*^_′ (*w*_7_, *o*_7_) < 1. Since word *w*_7_ appeared only in the late phase context Ω = {*w*_1_, *o*_1_, *w*_7_, *o*_7_} and object *o*_1_ was not displayed in the testing stage, we must conclude that the participants produced spurious associations between words and objects that never appeared together in a context. Our algorithm accounts for these associations through Equation (4) in the case of new words and, more importantly, through eqs. (9) and (13) due to the effect of the information efficiency factor α_*t*_ (*w*_*i*_). The experimental data is well described only in the narrow range α_0_ ∈ [0.85, 0.9].

**Figure 8:**
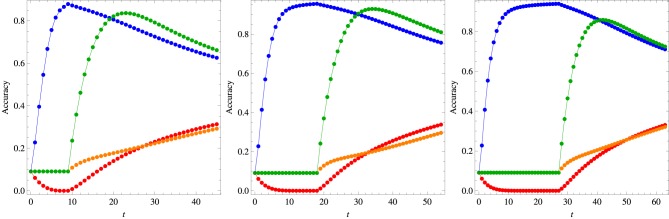
**Knowledge development for the model parameters that best fit the results of the mutual exclusivity experiments summarized in Figures 5, 6 in the case the late phase of the training process comprises 6 repetitions of each word**. The symbol colors follow the convention used in those figures, i.e., the blue symbols represent the confidence on association (*w*_1_, *o*_1_), the red symbols on association (*w*_1_, *o*_7_), the green symbols on association (*w*_7_, *o*_1_) and the orange symbols on association (*w*_7_, *o*_1_). The left panel shows the results for 3 repetitions of *w*_1_ in the early training phase (α_0_ = 0.85, χ = 0.25, β = 0.95), the middle panel for 6 repetitions (α_0_ = 0.85, χ = 0.45, β = 0.99) and the right panel for 9 repetitions (α_0_ = 0.85, χ = 0.4, β = 0.95). For each trial *t* the symbols represent the average over 10^5^ realizations of the learning process.

Figure 8 exhibits the developmental timeline of the cross-situational learning history of the algorithm with the optimal set of parameters (see the figure caption) for the three different training conditions in the early training phase. This phase is characterized by the steady growth of the confidence on the association (*w*_1_, *o*_1_) (blue symbols) accompanied by the decrease of the confidence on association (*w*_1_, *o*_7_) (red symbols). As the word *w*_7_ does not appear in the early training phase, the confidences on its association with any object remain constant corresponding to the accuracy value 1/11 (we recall that *o*_1_ is left out of the display in the testing stage). The beginning of the late training stage is marked by a steep increase of the confidence on the association (*w*_7_, *o*_7_) (green symbols) whereas the confidence on (*w*_1_, *o*_1_) decreases gradually. A similar gradual increase is observed on the confidence on the association (*w*_7_, *o*_1_) (orange symbols). As expected, for large *t* all confidences presented in this figure tend to the same value, since the words *w*_1_ and *w*_7_ always appear together in the context Ω = {*w*_1_, *o*_1_, *w*_7_, *o*_7_}. Finally, we note that this developmental timeline is qualitatively similar to that produced by the algorithm proposed by Kachergis et al. (2012).

## 5. Discussion

The chief purpose of this paper is to understand and model the mental processes used by human subjects to produce their word-object mappings in the controlled cross-situational word-learning scenarios devised by Yu and Smith (2007) and Kachergis et al. (2009, 2012). In other words, we seek to analyze the psychological phenomena involved in the production of those mappings. Accordingly, we assume that the completion of that task requires the existence of two cognitive abilities, namely, the associative capacity to create and reinforce associations between words and referents that co-occur in a context, and the non-associative capacity to infer word-object associations based on previous learning events, which accounts for the mutual exclusivity principle, among other things. In order to regulate the effectiveness of these two capacities we introduce the parameters χ ∈ [0, 1], which yields the reinforcement strength, and β ∈ [0, 1], which determines the inference strength.

In addition, since the reinforcement and inference processes require storage, use and transmission of past and present information (coded mainly on the values of the confidences *P*_*t*_ (*w*_*i*_, *o*_*j*_)) we introduce a word-dependent quantity α_*t*_(*w*_*i*_) ∈ [0, 1] which gauges the impact of the confidences at trial *t* − 1 on the update of the confidences at trial *t*. In particular, the greater the certainty about the referent of word *w*_*i*_, the greater the relevance of the previous confidences. However, there is a baseline information gauge factor α_0_ ∈ [0, 1] used to process words for which the uncertainty about their referents is maximum. The adaptive expression for α_*t*_(*w*_*i*_) given in Equation (7) seems to be critical for the fitting of the experimental data. In fact, our first choice was to use a constant information gauge factor (i.e., α_*t*_(*w*_*i*_) = α ∀*t*, *w*_*i*_) with which we were able to describe only the experiments summarized in Figures 1, 4 (data not shown). Note that a consequence of prescription (7) is that once the referent of a word is learned with maximum confidence (i.e., *P*_*t*_(*w*_*i*_, *o*_*j*_) = 1 and *P*_*t*_(*w*_*i*_, *o*_*k*_) = 0 for *o*_*k*_ ≠ *o*_*j*_) it is never forgotten.

The algorithm described in Section 3 comprises three free parameters χ, β and α_0_ which are adjusted so as to fit a representative selection of the experimental data presented in Kachergis et al. (2009, 2012). A robust result from all experiments is that the baseline information gauge factor is in the range 0.7 < α_0_ < 1. Actually, the fast mapping experiments narrow this interval down to 0.85 < α_0_ < 0.9. This is a welcome result because we do not have a clear-cut interpretation for α_0_—it encompasses storage, processing and transmission of information—and so the fact that this parameter does not vary much for wildly distinct experimental settings is evidence that, whatever its meaning, it is not relevant to explain the learning strategies used in the different experimental conditions. Fortunately, this is not the case for the two other parameters χ and β.

For instance, in the fast mapping experiments discussed in Subsection 4.3 the best fit of the experimental data is achieved for β ≈ 1 indicating thus the extensive use of mutual exclusivity, and inference in general, by the participants of those experiments. Moreover, in that case the best fit corresponds to a low (but non-zero) value of χ, which is expected since for contexts that exhibit two associations (*C* = 2) only, most of the disambiguations are likely to be achieved solely through inference. This contrasts with the experiments on variable word sampling frequencies discussed in Subsection 4.1, for which the best fit is obtained with intermediate values of β and χ so the participants' use of reinforcement and inference was not too unbalanced. The contextual diversity experiment of Subsection 4.2, in which the words are segregated in two isolated groups of 12 and 6 components, offers another extreme learning situation, since the best fit corresponds to χ ≈ 1 and β ≈ 0 in that case. To understand this result, first we recall that most of the participants' errors were due to misassignments of referents belonging to the same group of the test word, and those confidences were strengthened mainly by the reinforcement process. Second, in contrast to the inference process, which creates and strengthens spurious intergroup associations via Equation (12), the reinforcement process solely weakens those associations via Equation (9). Thus, considering the learning conditions of the contextual diversity experiment it is no surprise that reinforcement was the participants' choice strategy.

It is interesting to note that the optimal set of parameters that describe the fast mapping experiments (see Figures 5–8) indicate that there is a trade-off in the values of those parameters, in the sense that high values of the inference parameter β require low values of the reinforcement parameter χ. Since this is not an artifact of the model which poses no constrain on those values (e.g., they are both large for small α_0_), the trade-off may reveal a limitation on the amount of attentional resources available to the learner to distribute among the two distinct mental processes.

Our results agree with the findings of Smith et al. (2011) that participants use various learning strategies, which in our case are determined by the values of the parameters χ and β, depending on the specific conditions of the cross-situational word-learning experiment. In particular, in the case of low within-trial ambiguity those authors found that participants generally resorted to a rigorous eliminative approach to infer the correct word-object mapping. This is exactly the conclusion we reached in the analysis of the fast mapping experiment for which the within-trial ambiguity takes the lowest possible value (*C* = 2).

Although the adaptive learning algorithm presented in this paper reproduced the performance of adult participants in cross-situational word-learning experiments quite successfully, the deterministic nature of the algorithm hindered somewhat the psychological interpretation of the information gauge factor α_*t*_(*w*_*i*_). In fact, not only learning and behavior are best described as stochastic processes (Atkinson et al., 1965) but also the modeling of those processes requires (and facilitates) a precise interpretation of the model parameters, since they are introduced in the model as transition probabilities.

### Conflict of interest statement

The authors declare that the research was conducted in the absence of any commercial or financial relationships that could be construed as a potential conflict of interest.
